# The effect of in-bed cycling combined with high flow nasal cannula treatment on arterial oxygen and respiratory dynamics in patients with severe respiratory failure: A retrospective study

**DOI:** 10.12669/pjms.40.8.9471

**Published:** 2024-09

**Authors:** Xiaoyan Wang, Jiapo Zhang, Yang Jiang, Jie Liu, Deyuan Huo

**Affiliations:** 1 Xiaoyan Wang Department of Thoracic Surgery, Xiamen University Institute of Chest and Lung Disease, Xiang’an Hospital of Xiamen University, School of Medicine, Xiamen University, Xiamen, Fujian Province, P.R. China; 2 Jiapo Zhang Department of Emergency Medicine, Xiang’an Hospital of Xiamen University, School of Medicine, Xiamen University, Xiamen, Fujian Province, P.R. China; 3 Yang Jiang Department of Nursing, Xiang’an Hospital of Xiamen University, School of Medicine, Xiamen University, Xiamen, Fujian Province, P.R. China; 4 Jie Liu Department of Critical Medicine, The Second Affiliated Hospital of Harbin Medical University, Harbin, Heilongjiang Province, P.R. China; 5 Deyuan Huo Department of Critical Medicine, The Second Affiliated Hospital of Harbin Medical University, Harbin, Heilongjiang Province, P.R. China

**Keywords:** In bed cycling, High flow nasal canula, Arterial oxygen, Respiratory failure, Respiratory dynamics

## Abstract

**Objective:**

To assess the effects of in-bed cycling (IBC) combined with high flow nasal cannula (HFNC) on arterial oxygen and respiratory dynamics in patients with severe respiratory failure (RF).

**Methods:**

We retrospectively collected clinical data of 103 patients with severe RF, admitted to the intensive care unit (ICU) of The Second Affiliated Hospital of Harbin Medical University from March 2021 to March 2023. Among them, 50 patients had HFNC alone (control group), and 53 patients did IBC in addition to HFNC (observation group). We compared arterial oxygen index, lung function, respiratory dynamics, and clinical efficacy between the two groups.

**Results:**

There was no significant difference in the basic data between the two groups (*P*>0.05). After the treatment, the improvement of the partial pressure of oxygen (PaO_2_), PaO_2_/fraction of inspired oxygen (FiO_2_), arterial oxygen saturation (SaO_2_), and oxygen delivery (DO_2_) in the observation group was significantly better than that in the control group (*P*<0.05). After the treatment, the improvement of lung function in the observation group was better than that in the control group (*P*<0.05). After the treatment, the end expiratory pulmonary pressure (P_tp-ee_) and driving pressure (△P_tp_) levels in the observation group were significantly higher, and the duration of ICU hospitalization and the incidence of ICU-acquired weakness(ICU-AW) were significantly lower than those in the control group (*P*<0.05).

**Conclusions:**

IBC combined with HFNC can significantly improve arterial oxygen levels, lung function, and respiratory dynamics in patients with severe RF. IBC in combination with HFNC is associated with shorter stay time in the ICU, reduced of ICU-acquired weakness, and better physical recovery of patients.

## INTRODUCTION

Respiratory failure (RF) is characterized by the failure of lungs to maintain an appropriate gas exchange, leading to hypoxia or carbon dioxide retention and to physiological and metabolic disorders.^1^ The incidence of RF depends primarily on the cause of failure, and it was reported to be 1.4 to 9.5 cases per 100,000 children and adolescents per year worldwide, with estimated mortality over 24%-34%.^2^ RF is a critical life-threatening condition that requires immediate treatment.^1^,^2^

High flow nasal cannula (HFNC) is a method of supplemental oxygen therapy that is commonly used in ICU setting and can significantly improve hypoxia symptoms and reduce mortality.^3^ However, ICU patients may develop ICU-acquired weakness (ICU-AW), which hampers their recovery.^4^,^5^ Clinical studies have confirmed that early rehabilitation exercise can reduce the occurrence of ICU-AW, and promote the recovery of limb muscle strength and body function.^5^,^6^

In-bed cycling (IBC) is a novel modality that allows early initiation of physical activity in critically ill patients under mechanical ventilation.^7^ Multiple studies have confirmed that IBC exercise can slow down muscle atrophy, improve muscle strength, reduce complications caused by bed rest, and have a positive therapeutic effect on patients undergoing mechanical ventilation.^8^-^10^ However, IBC combined with HFNC on arterial oxygen and respiratory dynamics in patients with severe RF has not been explored in depth and few publication on it.

In recent years, our hospital has adopted IBC combined with HFNC treatment for patients with severe RF. This study aimed to review the collected data and to assess the effects of IBC combined with HFNC on arterial oxygen and respiratory dynamics in this group of patients.

## METHODS

Medical data of 103 patients with severe RF admitted to the ICU of The Second Affiliated Hospital of Harbin Medical University from March 2021 to March 2023 were retrospectively selected. Among them, 50 patients who received simple oxygen supplementation by HFNC comprised the control group, and 53 patients who performed IBC combined with HFNC were set as the observation group.

### Ethical Approval:

The ethic committee of the Second Affiliated Hospital of Harbin Medical University approved this study on November 29, 2023, case number KY2023-159.

### Inclusion criteria:

- Meets the clinical diagnostic criteria for severe RF.^11^
- Age ≥ 18 years old.
- Complete clinical data.

### Exclusion criteria:

- Individuals with severe organic lesions such as heart, liver, lungs, kidneys, and disorders of the hematopoietic and coagulation systems.
- Individuals with muscle weakness, fractures, and neurological damage.
- Patients with indications for emergency organ intubation.
- Pregnant and lactating women.

### HFNC

The instruments used for patients in the control group were manufactured by Fisher and Paykel Healthcare with the following parameters: gas flow rate at 50 L/minute with the initial FiO_2_ at 100%. Then, the oxygen concentration was adjusted to make the PaO_2_ equal to 92%. Treatment time was ≥ 48 hours. After the initial period of 48 hours, the settings were adjusted based on the patient’s condition.

### IBC

The bed bicycle model used was KFO3 Series from Shanghai Mei Health Rehabilitation Equipment Co. This model has passive and active modes. The speed per minute was set in the passive mode, and the patient controlled it in the active mode. The specific procedures were the following: the attending physician evaluated patients who met the conditions for early rehabilitation activity and instructed them how to use the bicycle. Patients with lower limb muscle strength below grade-3 used the passive mode. On the first day of the activity, the speed was set to 10 times/ minutes) in the passive mode. If the heart rate and blood pressure changes during the activity were smaller than 20% of the base levels, the speed was increased by 3 ~ 5 times/minute the next day (the maximum speed was 20 times/minutes).

Patients with lower limb muscle strength above level-4 used the active mode, in which they could independently step on the bed bicycle for activities. If the rotational speed in the active mode was more than 20 times/minute, and the heart rate and blood pressure changes remained below 20% of the basic level (the patient was not making any effort), the patient was instructed to extend the activity time on the next day. If the rotational speed was less than eight times/ minutes) in the active mode and the patient was not able to modify it after communicating with them, the following rehabilitation exercises were continued in the passive mode.

The patients were offered training in bed (active or passive mode) twice a day in the morning and afternoon for 20-30 minutes each time. Upper limb passive joint activities (range of motion, [ROM] exercises) were performed on the upper limbs while the patients were using the bed bicycle for the lower limbs. The upper limb passive joint activity exercises included elbow flexion/extension 0° to 90°, wrist palmar flexion/extension 0° to 80°, and forearm pronation/supination 0° to 80°. These exercises were repeated 10-15 times, and each activity lasted for 15-30 minutes. We discontinued the exercise whenever patients developed discomfort during a session.

### Collected indicators and efficacy criteria:

- Arterial blood oxygen indexes, including PaO_2_, carbon dioxide (PaCO_2_), PaO_2_/FiO_2_, SaO_2_, and DO_2_, was measured using Stat profile pHox blood gas analyzer (NOVA company; USA).
- Lung function indexes, including forced vital capacity (FVC), maximum voluntary ventilation (MVV), forced expiratory volume in the first second (FEV_1_), and airway resistance (Raw) were measured at various time points using S-980A lung function tester (Shanghai Hanfei Medical Equipment Co., Ltd.).
- Respiratory mechanics indicators, including respiratory rate (RR), end inspiratory transpulmonary pressure (P_tp-ei_), P_tp-ee_, and P_tp_ were measured using Ventrak respiratory function monitor (Novamatrix, USA).
- Clinical efficacy indicators, including ICU hospitalization time and ICU-AW incidence rate.

### Statistical Analysis:

Data were entered into Microsoft Excel and analyzed using SPSS version 22.0 (IBM Corp, Armonk, NY, USA). Continuous variables were reported as mean and standard deviation (SD) using t-tests. The counting data were represented by the number of use cases using chi square test. P<0.05 indicated statistically significant difference. PRISM 8.0 software (GraphPad, San Diego, USA) was used to plot changes in arterial blood oxygen and respiratory dynamics indicators.

## RESULTS

A total of 103 patients met the conditions for this study, 50 patients in the control group (HFNC alone) and 53 patients in the observation group (IBC + HFNC). There was no significant difference in the basic data between the two groups (*P*>0.05) (Table-I).

**Table-I:** Comparison of basic information between two groups.

Group	Gender (male/female)	Age (years)	BMI (kg/m²)	APACHE II (score)
Control group (n=50)	28/22	58.26±7.97	24.25±3.04	17.98±3.38
Observation group (n=53)	32/21	59.04±10.18	23.82±3.12	18.40±3.43
χ^2^/t	0.203	-0.43	0.707	-0.62
P	0.653	0.668	0.481	0.536

***Note:*** BMI = body mass index; APACHE II = acute physiology and chronic health evaluation II.

**Fig.1:**
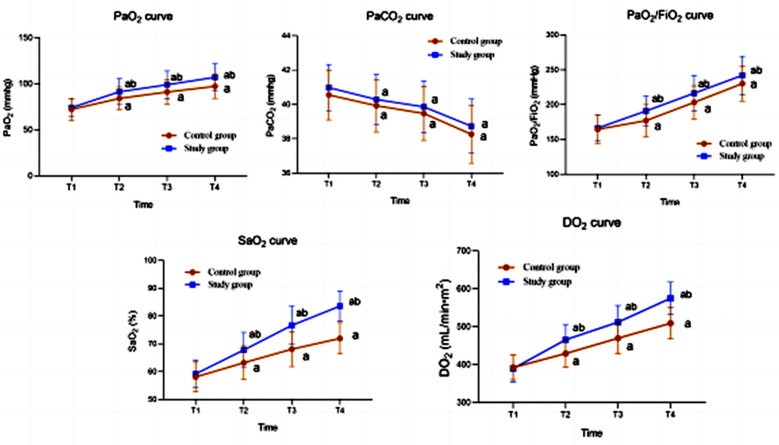
Blood oxygen index curve of the two groups. Compared with before treatment in the same group, ^a^*P*<0.05; Compared with the control group, ^b^*P*<0.05; T1: before treatment; T2: 6-hours after treatment; T3: 24-hours after treatment; T4: 48-hours after treatment.

Before the treatment, there was no significant difference in the blood oxygen indicators between the two groups (*P*>0.05). After 6, 24, and 48 hours of treatment, both groups showed significant improvements in PaO_2_, PaCO_2_, PaO_2_/FiO_2_, SaO_2_, and DO_2_ compared to before the treatment (*P*<0.05). PaO_2_, PaO_2_/FiO_2_, SaO_2_, and DO_2_ in the observation group were significantly higher than those in the control group at the same time points (*P*<0.05) (Fig.1).

There was no significant difference in lung function indicators between the two groups before the treatment (*P*>0.05). After the treatment, the lung function indicators of both groups were significantly improved, and were significantly better in the observation group compared to the control group (*P*<0.05) (Table-II).

**Table-II:** Comparison of lung function indicators between two groups.

Group	Time	FVC (L)	MVV (L)	FEV1 (%)	Raw[cmH_2_O•(L•s)^-1^]
Control group (n=50)	Before treatment	1.65±0.22	61.14±5.67	40.4±4.04	18.22±3.12
	After treatment	2.22±0.31^a^	81.3±7.34^a^	45.74±5.52^a^	10.32±2.97^a^
Observation group (n=53)	Before treatment	1.61±0.25	62.26±5.54	39.77±4.84	17.74±3.09
	After treatment	3.85±0.36^ab^	92.32±8.91^ab^	49.04±6.01^ab^	6.3±2.75^ab^

***Note:*** Compared with before treatment in this group,

a P<0.05; Compared with the control group, ^b^P<0.05. FVC = forced vital capacity; MVV = maximum voluntary ventilation; FEV_1_ = forced expiratory volume in the first second; Raw = airway resistance.

**Fig.2:**
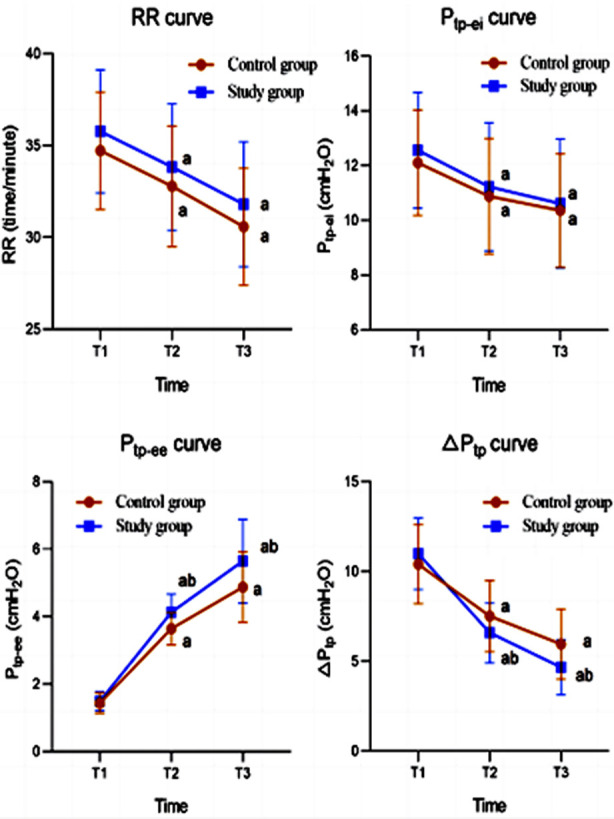
Respiratory dynamics index curve of the two groups. Compared with before treatment in the same group, ^a^*P*<0.05; Compared with the control group, ^b^*P*<0.05; T1: before treatment; T2: 30-minutes after treatment; T3: 60-minutes after treatment.

Before the treatment, there was no significant difference in the respiratory dynamics indicators between the two groups (*P*>0.05). After 30 and 60 minutes of treatment, both groups showed significant improvement in respiratory dynamics indicators (*P*<0.05). P_tp-ee_ and △P_tp_ levels in the observation group were significantly higher than those in the control group at the same time points (*P*<0.05) (Fig.2). After the treatment, the ICU hospitalization time and ICU-AW incidence in the observation group were significantly lower than those in the control group (*P*<0.05) (Table-III).

**Table-III:** Comparison of clinical efficacy between two groups.

Group	n	ICU hospitalization (day)	ICU-AW (%)
Control group	50	12.74±1.69	25 (50.0%)
Observation group	53	11.13±1.04*	13 (24.5%)^*^
t/χ^2^		5.859	7.170
P		<0.001	0.007

***Note:*** ICU-AW = ICU-acquired weakness.

## DISCUSSION

The results of this study show that compared with HFNC alone, IBC combined with HFNC treatment can significantly improve blood oxygen metabolism, lung function, and respiratory dynamics in patients with severe RF. IBC combined with HFNC was able to shorten the ICU stay time of patients with RF and reduce the incidence of ICU-AW. Our results confirm that ICU RF patients would benefit from engaging in early active/passive bed movements and ambulatory activities. It also indicates that IBC combined with HFNC treatment can improve lung function, oxygen metabolism, and respiratory dynamics in RF patients, and reduce lung driving pressure.^7^,^12^,^13^

The clinical significance of this effect lies in the reduction of driving pressure, which can alleviate lung tissue damage caused by lung ventilation, reduce respiratory muscle work, alleviate respiratory muscle fatigue, and ultimately reduce patient mortality.^7^,^12^ Nickels *et al*.^7^ showed that when started in the early stages of critical illness, IBC for critically ill patients is safe and feasible in a tertiary ICU environment in Australia, and allows to maintain muscle mass and improve recovery. Nickels *et al*.^12^ also showed that early IBC has significant advantages in maintaining muscle and improving body function in ICU patients. Our study focused on assessing the changes in blood oxygen, lung function, and respiratory dynamics before and after IBC treatment in patients with severe RF, and the results are consistent with those of Wang *et al*.^13^

HFNC mode of oxygen supplementation heats and humidifies the inhaled gases, alleviates small airway spasms, improves ciliary motor function, and promotes the clearance of respiratory secretions.^4^ Additionally, it can create positive end-expiratory pressure in the respiratory tract, promote alveolar recruitment, reduce anatomical dead space, and increase end-expiratory volume.^4^,^14^ Therefore, HFNC was used as a baseline support treatment for all patients in this study. Previous reports showed that early rehabilitation exercises for patients with RF can effectively prevent ventilator-related complications and promote physical recovery.^15^ The IBC implemented in this study went from passive to active, with a duration of time from short to long, and intensity from weak to strong, following the principle of gradual rehabilitation exercise for patients.^14^,^15^

The results of this study indicate that IBC combined with HFNC treatment can improve lung ventilation and ventilation ability in RF patients and increase blood oxygen metabolism levels. This is mainly related to the fact that HFNC can quickly improve patient oxygenation status, provide positive end-expiratory pressure at 3-7 cmH_2_O levels, promote end-expiratory alveolar opening and gas exchange.^14^,^16^ In addition, HFNC can be adjusted according to patient needs, and the oxygen concentration can be controlled between 21% and 100%, providing personalized respiratory support for different patients. At the same time, high-speed airflow can be used to flush the dissected invalid cavities, increasing alveolar ventilation volume, thus positively improving lung ventilation and ventilation function.^4^,^14^,^16^ IBC not only improves the endurance of both lower limb muscles, but also strengthen intercostal muscles, diaphragm, and abdominal muscles, promoting inhalation and forced exhalation, and facilitating recovery.^7^,^12^,^15^

Lung function, blood oxygen metabolism, and respiratory dynamics are all methods for evaluating respiratory function.^17^,^18^ The results of our study showed that after the treatment, lung function indicators, oxygen metabolism indicators, and respiratory dynamics indicators in both groups were significantly improved compared to before the treatment, and were significantly better in patients who were engaged in IBC in addition to HFNC oxygen supplementation, compared to patients with HFNC alone.

Eggmann *et al*.^19^ found that frailty at ICU discharge is associated with short-term dysfunction and prolonged hospitalization, and that early and effective rehabilitation exercises can maximize the strength and endurance of the main muscles in critically ill patients and maintain joint range of motion. Studies show that rehabilitation exercises shorten the time of mechanical ventilation time, ICU occupancy time, reduce the occurrence of ICU-AW, promote disease recovery and the recovery of body functional status.^20^-^22^ Yu et al. also reported that IBC with passive joint movement can reduce the duration of mechanical ventilation, ICU stay and incidence of ICU-AW as well as improve their ability to live independently.^23^

The results of our study also indicate that IBC combined with HFNC can shorten the mechanical ventilation time and ICU stay time of RF patients, reduce the incidence of ICU-acquired weakness and promote patient recovery, which is consistent with the previous research.^20^-^23^ From this, it can be seen that this study confirms that early active/passive bed movement and getting out of bed activities in ICU patients can reduce the incidence of acquired weakness in ICU and promote patient recovery.

### Limitations:

Firstly, the assessment was conducted in a single medical ICU, so the results may not be generalizable to other ICUs or patient groups. Secondly, incomplete medical records were excluded, which may have led to selection bias. Thirdly, neither group was randomly assigned, and therefore, baseline information may be imbalanced and biased. Finally, the follow-up time was short and it was not possible to assess the prognosis. Further high-quality research is needed to verify the conclusions of this study.

## CONCLUSION

IBC combined with oxygen supplementation via HFNC can significantly improve blood oxygen metabolism, lung function, and respiratory dynamics in patients with severe RF compared to HFNC alone. IBC/HFNC regimen can shorten the ICU stay time, reduce the incidence of ICU-AW, and promote physical recovery of the patients with severe RF.

### Authors’ Contributions:

**XW:** Conceived and designed the study.

**XW JZ, YJ, JL and DH:** Collected the data and performed the analysis.

**XW:** Was involved in the writing of the manuscript and is responsible for the integrity of the study.

All authors have read and approved the final manuscript.
